# A mesh generation and machine learning framework for *Drosophila* gene expression pattern image analysis

**DOI:** 10.1186/1471-2105-14-372

**Published:** 2013-12-28

**Authors:** Wenlu Zhang, Daming Feng, Rongjian Li, Andrey Chernikov, Nikos Chrisochoides, Christopher Osgood, Charlotte Konikoff, Stuart Newfeld, Sudhir Kumar, Shuiwang Ji

**Affiliations:** 1 Department of Computer Science, Old Dominion University, Norfolk, VA 23529, USA; 2 Department of Biological Sciences, Old Dominion University, Norfolk, VA 23529, USA; 3 Center for Evolutionary Medicine and Informatics, Biodesign Institute, Arizona State University, Tempe, AZ 85287, USA; 4 School of Life Sciences, Arizona State University, Tempe, AZ 85287, USA; 5 Center of Excellence in Genomic Medicine Research, King Abdulaziz University, Jeddah, Saudi Arabia

## Abstract

**Background:**

Multicellular organisms consist of cells of many different types that are established during development. Each type of cell is characterized by the unique combination of expressed gene products as a result of spatiotemporal gene regulation. Currently, a fundamental challenge in regulatory biology is to elucidate the gene expression controls that generate the complex body plans during development. Recent advances in high-throughput biotechnologies have generated spatiotemporal expression patterns for thousands of genes in the model organism fruit fly *Drosophila melanogaster*. Existing qualitative methods enhanced by a quantitative analysis based on computational tools we present in this paper would provide promising ways for addressing key scientific questions.

**Results:**

We develop a set of computational methods and open source tools for identifying co-expressed embryonic domains and the associated genes simultaneously. To map the expression patterns of many genes into the same coordinate space and account for the embryonic shape variations, we develop a mesh generation method to deform a meshed generic ellipse to each individual embryo. We then develop a co-clustering formulation to cluster the genes and the mesh elements, thereby identifying co-expressed embryonic domains and the associated genes simultaneously. Experimental results indicate that the gene and mesh co-clusters can be correlated to key developmental events during the stages of embryogenesis we study. The open source software tool has been made available at http://compbio.cs.odu.edu/fly/.

**Conclusions:**

Our mesh generation and machine learning methods and tools improve upon the flexibility, ease-of-use and accuracy of existing methods.

## Background

Advances in sequencing and gene-prediction technologies have led to the discovery of virtually complete sets of protein-coding sequences in many model systems. In contrast, how these coding sequences are controlled by the regulatory sequences to transform a single cell, through cell division and differentiation, into a complex multicellular organism remains largely unknown. In multicellular organisms, one of the primary purposes of gene control is execution of the genomic regulatory code to generate complex body plans during development [1,2]. This process critically depends on the right gene being activated in the right cell (spatially) at the right time (temporally). Thus, analysis of spatiotemporal gene expression patterns provides a promising way for investigating the gene regulatory networks governing development.

In developmental biology, the fruit fly *Drosophila melanogaster* has long been established as a canonical model organism [3,4]. Recent advances in high-throughput *in situ* hybridization (ISH) technologies have allowed scientists to produce spatiotemporal expression patterns for thousands of genes in *Drosophila*[5-8]. This wealth of data creates opportunities for studying the developmental regulatory networks. However, the sheer volume and complexity of these data preclude the traditional practice of manual analysis and make automated methods essential [8-16].

In this work, we develop a set of ISH image computing and machine learning methods for the automated analysis of *Drosophila* gene expression pattern images. Specifically, we develop a mesh generation pipeline for mapping the expression patterns of many genes into the same geometric space [8]. This enables accurate comparative analysis of the spatial expression patterns of multiple genes and accounts for the differences in embryo morphology. We fit an ellipse to the boundary of each embryo using the least squares criterion. We then average the fitted ellipses for all images in the same stage range to obtain a generic ellipse. We automatically interpolate the boundary of this generic ellipse and use a Delaunay mesh method [17-20] to generate a triangulated mesh on this ellipse.

We accurately capture the morphology of each embryo by employing a systematic procedure to deform the generic, meshed ellipse to each individual embryo. We first establish correspondences between vertices on the generic ellipse and those on the fitted ellipses. Then the vertices on the fitted ellipses are deformed to the embryo boundary using the minimum distance criterion. Finally, the coordinates of all the other vertices are computed by solving an elastic finite element problem.

The mesh generation scheme allows us to organize the expression pattern images of many genes into a data matrix in which one dimension corresponds to genes and the other dimension corresponds to mesh elements as in the Genomewide-Expression-Maps (GEMs) [12,21]. To identify co-expressed embryonic domains and the associated genes, we develop a co-clustering formulation to cluster the mesh elements and the genes simultaneously. We formulate the co-clustering problem using a maximum likelihood formalism and employ an expectation-maximization algorithm to perform the parameter estimation.

We apply the mesh generation and co-clustering methods to a set of gene expression pattern images in the FlyExpress database [12]. Our results show that our methods generate co-expressed domains that overlap with many embryonic structures. In addition, these results show that the proposed methods yield gene clusters that are functionally more enriched than those discovered in prior studies. More importantly, we show that the mesh and gene co-clusters correlate strongly with key developmental events during the stages of embryogenesis under investigation.

## Methods

### Mesh generation

#### Requirements

Let *I*_1_,…,*I*_*m*_ be a list of embryo images. The goal of this module of the pipeline is to overlay each of the embryo images with a triangular mesh, such that all meshes have the same number of triangles and connectivity. For a given image, all triangles we create are of approximately the same size, in terms of their area. Let *a* stand for an upper bound on triangle area. Then all triangles in a single mesh which we construct have area slightly less than *a*. Let *M*_*j*_(*a*) be the mesh that we construct for image *I*_*j*_ that depends on area bound *a*. For simplicity we will omit the parameter *a* below.

More precisely, let *M*_*j*_ = (*V*_*j*_,*T*_*j*_), where *V*_*j*_ is the list of vertices and *T*_*j*_ is the list of triangles. Each vertex is defined by its two-dimensional coordinate, and each triangle is defined by a triple of vertex indices (*p*_1_,*p*_2_,*p*_3_), 1 ≤ *p*_1_,*p*_2_,*p*_3_ ≤ |*V*_*j*_|. These meshes are expected to satisfy the following requirements: 

• All of the *T*_*j*_ contain the same number of triangles, *i.e.*, |*T*_*j*_| = |*T*_*i*_| for *i*,*j* = 1,…,*m*.

• All of the *T*_*j*_ contain the same triples of vertex indices in the corresponding positions. As a result, we can omit the subscript and use *T* for all meshes *M*_*j*_, *j* = 1,…,*m*.

• All of the *V*_*j*_ contain the same number of vertices: |*V*_*j*_| = |*V*_*i*_| for *i*,*j* = 1,…,*m*.

• All vertices on the boundary of mesh *M*_*j*_ lie on the boundary of the embryo of image *I*_*j*_.

• Each triangle in *M*_*j*_ = *M*_*j*_(*a*) has area approximately equal to *a*.

• All vertices in *V*_*j*_ are geometrically close to the vertices in the corresponding positions in *V*_*i*_ for all *i*,*j* = 1,…,*m*, with respect to their location within an embryo.

#### Construction and meshing of the average ellipse

For each image *I*_*j*_, *j* = 1,…,*m*, we compute the parameters of the equation of the ellipse *E*_*j*_ that realizes the best fit to the boundary of the embryo in this image. We compute the best fitted ellipse using the least squares criterion to the set of the embryo’s boundary pixels. Then we average the parameters of all ellipses to obtain the average ellipse *E*^′^.

Given a value of *a*, we construct a mesh of *E*^′^. First, we use linear interpolation to approximate the boundary of *E*^′^, and then use a Delaunay mesh generator, Triangle [17], to mesh the interior of *E*^′^. Delaunay refinement is our meshing method of choice since it is backed by proven theoretical guarantees [18-20] that make it a push-button technology: its being able to guarantee termination with angle and area bounds allow for a guaranteed quality automatic pipeline.

We interpolate the boundary of *E*^′^ by performing the following steps. First, we calculate the side length *ℓ* of an equilateral triangle with area *a*. Then we use an iterative subdivision of the boundary of *E*^′^ with a set of vertices *v*_1_,…,*v*_*s*_ = *v*_0_ until all segment lengths |*v*_*i*-1_*v*_*i*_|, *i* = 1,…,*s* are approximately equal to *ℓ*. In other words, this is a uniform distribution of vertices with respect to the lengths of segments. The union of all these segments is a piecewise linear interpolation of the boundary of *E*^′^.

To tessellate the interior of *E*^′^, we use Triangle with the following parameters: 

• A planar straight line graph (PSLG) composed of the segments and the points interpolating the boundary of *E*^′^ plus one point in the center of *E*^′^. We instruct Triangle to preserve this PSLG and not to split the boundary segments, so that the discretization of the PSLG appears as a subgraph of the final mesh.

• The area bound *a* instructing Triangle to produce all triangles with areas bounded from above by *a*. Triangle starts with a coarse mesh and iteratively splits triangles until their areas fall below *a*, and therefore this is an approximate target area.

• An angle bound of 25° which instructs Triangle to enforce all angles in the final mesh to be 25° or above. Theoretically, Triangle guarantees only a minimum angle bound of 20.7° or below, however we found that in practice it can mesh an ellipse with a 25° angle bound, since it is a simple shape.

Let the mesh of the average ellipse be denoted as *M*^′^, and the list of radial angles corresponding to the subdivision vertices as $\theta_{1}^{\prime },\ldots ,\theta_{s}^{\prime }$.

#### Deformation of the mesh of the average ellipse

For each ellipse *E*_*j*_, we use the angles $\theta_{1}^{\prime },\ldots ,\theta_{s}^{\prime }$ to find the vertices that discretize the boundary of *E*_*j*_. Then we project these vertices onto the closest points from the boundary of the embryo in image *I*_*j*_. We define closeness in terms of the Euclidean distance, and use the Matlab’s Euclidean distance transform function to find the nearest boundary pixels simultaneously for all pixels in the image. Using the result of this function, we determine the required projections.

For each image *I*_*j*_, we deform the mesh *M*^′^, such that the boundary vertices of *M*^′^ assume the coordinates of the corresponding vertices (with respect to their radial ordering) on the boundary of the embryo in *I*_*j*_. The target coordinates of all the other vertices in *V*^′^ are computed by solving an elastic finite element problem [22]. As a result, the triangles of the generic mesh are deformed minimally and proportionally to their distance to the projected vertices on the boundary of the embryo in *I*_*j*_ and to the amount of the displacement at these boundary vertices.

### Simultaneous clustering of mesh elements and genes

For a mesh with *n* elements (triangles), we assume that the elements are numbered from 1 to *n* in an arbitrary but fixed order. Following [8], we extract the median of gray-level intensities from each mesh element and represent each image using an *n*-dimensional vector in which the *i*th component contains the median of intensities from the *i*th mesh element. Then the expression patterns of *m* genes can be encoded into a data matrix $A\in R^{m\times n}$, in which each row corresponds to a gene, and each column corresponds to a mesh element. Note that, to simplify the notation, we assume that the number of images and the number of genes are the same. When the expression pattern of a gene is captured by multiple images, we treat them separately.

In [8], two clustering methods are applied independently to identify clusters in the rows or the columns of the matrix *A*. In their case the row-wise (column-wise) clustering requires the rows (columns) in the same cluster to be similar with respect to all columns (rows). However, a set of genes might be co-expressed only at certain local domain of the embryo corresponding to a subset of mesh elements. To identify the co-expressed embryonic domains and the associated genes, we employ a co-clustering method to cluster the rows and columns of the data matrix *A* simultaneously. This generates co-clusters consisting of a subset of genes that are co-expressed at a subset of mesh elements. Note that entries of matrix *A* encode the expression intensities of genes and thus are nonnegative. An appealing property of our co-clustering method is that it is based on a probabilistic model and thus preserves the nonnegativity in the estimated parameters. It has been shown in [23] that a variant of this model consistently outperforms other methods that do not preserve nonnegativity.

#### A co-clustering formulation

In our co-clustering model, the matrix *A* is represented as a bipartite graph in which the two set of vertices correspond to the rows (genes) and columns (mesh elements), respectively, of the matrix *A*. The edge connecting the *i*th vertex in the first set to the *j*th vertex in the second set carries a weight of *A*_*ij*_. It follows that the adjacency matrix of the bipartite graph *W* can be expressed as

(1) $$W=\left[\begin{array}{cc} 0 & A\\ A^{T} & 0 \end{array}\right],$$

where the vertices in one set is ordered before vertices in the other set.

We assume that the adjacency matrix *W* of the bipartite graph can be approximated by

(2) $$W\approx H\tilde{H},$$

where

(3) $$H=\left[\begin{array}{cc} P & 0\\ 0 & Q \end{array}\right]\in R^{\left(m+n)\times (2c\right)},$$

(4) $$\tilde{H}=\left[\begin{array}{cc} 0 & Q^{T}\\ P^{T} & 0 \end{array}\right]\in R^{\left(2c)\times (m+n\right)},$$

*c* is the number of co-clusters, $P\in R^{m\times c}$ denotes the row cluster indicator matrix, and $Q\in R^{n\times c}$ denotes the column cluster indicator matrix. It follows that

(5) $$H\tilde{H}=\left[\begin{array}{cc} 0 & PQ^{T}\\ {\left(PQ^{T}\right)}^{T} & 0 \end{array}\right],$$

which matches the structure of *W* in Eq. (1).

Following [24], we assume that the data in *A* are generated via a multinomial distribution. This gives rise to the following log likelihood function of observing the adjacency matrix *W*:

(6) $$\begin{array}{l} L=\log P(W|H\tilde{H})\\ \;=2\overset{m}{\underset{i=1}{\sum }}\overset{n}{\underset{j=1}{\sum }}\;A_{\text{ij}}\log{\left(PQ^{T}\right)}_{\text{ij}}. \end{array}$$

It can be shown [24] that maximizing the log likelihood in Eq. (6) is equivalent to minimizing the divergence loss of the approximation in Eq. (2).

#### An EM algorithm

We use an EM algorithm to maximize the log likelihood in Eq. (6). In the following, variables with hat are used to denote the values obtained from the previous iteration. In the E-step, we compute the expectation as

(7) $$\phi_{\text{ijk}}=\frac{{\hat{P}}_{\text{ik}}{\hat{Q}}_{\text{jk}}}{{\left(\hat{P}{\hat{Q}}^{T}\right)}_{\text{ij}}}.$$

In the M-step, we maximize the expectation of log likelihood with respect to (*Φ*)_*ijk*_ = *ϕ*_*ijk*_

(8) $$\begin{array}{l} E_{\Phi }[L]=2\times \underset{\text{ijk}}{\sum }\;\phi_{\text{ijk}}A_{\text{ij}}\;\log\left(P_{\text{ik}}Q_{\text{jk}}\right). \end{array}$$

We impose the following normalization constraints to facilitate a probabilistic interpretation of the co-clustering results:

$$\overset{m}{\underset{i=1}{\sum }}P_{\text{ik}}=1,\;\overset{n}{\underset{j=1}{\sum }}Q_{\text{jk}}=1.$$

By using Lagrange multipliers for these constraints, it can be shown that the following update rules will monotonically increase the expected log likelihood defined in Eq. (8), thereby converging to a locally optimal solution [24]:

$$\begin{array}{l} P_{\text{ik}}\leftarrow 2\times \underset{j}{\sum }\frac{{\hat{P}}_{\text{ik}}{\hat{Q}}_{\text{jk}}A_{\text{ij}}}{{\left(\hat{P}{\hat{Q}}^{T}\right)}_{\text{ij}}},\\ Q_{\text{jk}}\leftarrow 2\times \underset{i}{\sum }\frac{{\hat{P}}_{\text{ik}}{\hat{Q}}_{\text{jk}}A_{\text{ij}}}{{\left(\hat{P}{\hat{Q}}^{T}\right)}_{\text{ij}}}. \end{array}$$

The results are then normalized such that ∑_i_ *P*_*ik*_ = 1 and ∑_j_ *Q*_*jk*_ = 1. The E-step and M-step are repeated until a locally optimal solution is obtained. Then the matrices *P* and *Q* can be used as row and column co-cluster indicator matrices, respectively, to obtain soft co-clustering results. A variant of this method has been shown to compare favorably with other approaches on a variety of data sets [23].

## Related work

**Figure 1:**
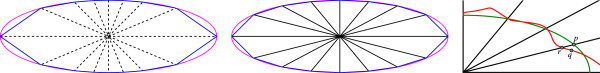
**Left: Subdivision of an ellipse (pink) based on equal radial angles (dashed black lines) leads to inaccurate boundary interpolation (blue).** **Center:** A more accurate subdivision (solid black lines) based on equal lengths of interpolating segments. **Right:** Euclidean projection (*q*) from a point (*p*) on the ellipse (green) onto the boundary of the embryo (red) is more accurate than a projection along a radial line (*r*).

Our work on mesh generation is motivated by the prior work in [8]. However, there are some substantial differences between our approach and the prior method. Besides the expanded analysis based on meshes with a range of triangle sizes, for a given triangle size *a* our methodology also offers a number of significant improvements in the accuracy of capturing embryo shapes. Frise *et al.*[8] define *E*^′^ as a predetermined ellipse of axial ratio 4:2, while we compute *E*^′^ from the actual embryo shapes. As a result, we make sure that *E*^′^ is close to the particular set of shapes, since different sets of shapes can have different average ellipses. Frise *et al.*[8] discretize the boundary of *E*^′^ based on approximately equal radial angles, while our discretization is based on approximately equal edge lengths. See Figure 1 (left and center) for an illustration. Frise *et al.*[8] project the discretization vertices from *E*_*j*_ onto the actual boundary of the embryo along the radial lines emanating from the center of *E*_*j*_, while we choose the closest points based on Euclidean distance. See Figure 1 (right) for an illustration.

Our work is related to the seminal work in [25], where the Gaussian mixture models (GMM) were applied to generate co-expression domains for the purpose of image comparison. Our work is different from [25] in both its objectives and approaches. In [25], image pixels were considered directly as the basic elements of modeling while we use triangulated mesh to warp and discretize the embryos in order to account for the shape and morphological variations. It has been shown in prior work [8] that the use of mesh leads to biologically significant results. In addition, GMM was used to cluster the pixels in [25], while we use a co-clustering method to co-cluster the mesh elements and the genes simultaneously. Since each domain is expected to be defined by only a subset of genes in the genome, co-clustering aims at identifying the domains and the associated genes simultaneously. As shown by our experimental results, co-clustering leads to more significant results.

## Results and discussion

We evaluate the proposed computational methods on a set of gene expression pattern images retrieved from the FlyExpress database [12]. This database contains genome-wide, two-dimensional, standardized images obtained from multiple sources, including the Berkeley *Drosophila* Genome Project [5]. Other databases provide three-dimensional images with higher resolution, but the data are not on the genome-scale [26]. Following [8], we focus on stages 4-6 and generate two data sets. The larger data set contains 2693 images capturing the expression patterns of 1881 genes, and the smaller one is a subset of 553 images corresponding to 365 genes with clearly defined expression boundaries. The images are preprocessed by a set of tools developed in [8] before they are tesselated with our mesh generation tools. We apply the proposed mesh generation method to convert a set of images into a data matrix in which the rows correspond to genes and the columns correspond to mesh elements. We apply the co-clustering method to compute co-clusters of genes and mesh elements. We first study the mesh clusters and gene clusters separately in Sections “Clustering of mesh elements” and “Clustering of genes”, respectively. We then correlate mesh and gene co-clusters with developmental events in Section “Co-clustering of mesh elements and genes.

### Clustering of mesh elements

**Figure 2:**
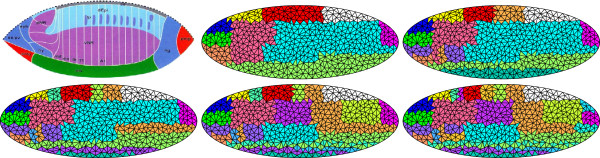
**Clusters of mesh elements when the number of clusters is varied from 10 to 30 with a step size of 5 (left to right, top to bottom) on stage 4-6 expression patterns.** The first figure in the first row shows the fate map of the blastoderm [27].

**Figure 3:**
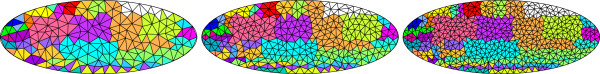
**Clusters of mesh elements when the number of co-clusters is set to 39 as in **[8]**, and when the number of mesh elements are set to 300, 600, and 1000 (left to right).** In these figures, colors are used to visualize clusters so that mesh elements in the same cluster are in the same color, and those in different clusters are in different colors.

The mesh elements represent localized spatial areas of the embryo, and can be used to discover distinct domains of developmental gene expression. We apply our mesh method to the data set of 553 stage 4-6 lateral embryos to gain insight into major developmental co-expression domains during this time. Co-clustering with different numbers of co-clusters is applied to the data matrix. Results are then mapped to the average ellipse and color-coded (Figures 2 and 3). To ensure that cluster boundaries are not the result of data processing artifacts, data is randomized at multiple points of the pipeline.

Figures 2 and 3 reveal the resulting clusters resemble the fate map of the developing embryo [27]. The clusters represent domains of high co-expression. They invariably form spatially contiguous regions, and are composed of rectangular shapes. Further, the cluster boundaries are largely parallel to the anterior/posterior (A/P) and dorsal/ventral (D/V) axes of the embryo. As the number of co-clusters is increased (Figure 2), the rectangular cluster shape is often retained, with larger clusters subdivided into smaller ones. In our data set, this subdivision of clusters often occurred at the far A/P and D/V regions of the embryo. These increased subdivisions correlate with major developmental events during stages 4-6 of *Drosophila* embryogenesis [4,27]. Signals along the A/P and D/V axes drive this pattern formation [3]. During Stage 6 gastrulation begins, and the ventral and cephalic furrows form. Looking back at the clusters, we see a greater proportion of subdivisions along where these furrows form in the developing embryo. The general clustering patterns remain the same while the cluster boundaries become smoother as the number of mesh elements increases (Figure 3).

### Clustering of genes

Co-clustering of the data matrix leads to clusters of genes. We use gene ontology (GO) [28] to evaluate the gene clusters and compare the results with those reported in [8]. Our gene clusters are the combined results of the mesh generation and co-clustering methods. Hence, we evaluate the effects of these two methods separately.

First, we compare our mesh generation method with the approach described in [8]. We apply both methods to the set of 553 images, yielding two data matrices. We then apply the co-clustering method with different numbers of co-clusters to these two data matrices. Since the same co-clustering method is used for both data matrices, the differences in the results should be contributed by differences in the mesh generation methods. We use the hypergeometric distribution to compute enriched GO terms [29] in order to evaluate the gene clusters generated from these two data matrices. The numbers of terms with *p*-values less than 0.001 are reported in Table 1. We can see that these two methods give similar numbers of biological process terms when the number of clusters is relatively small (30-35). However, as the number of cluster increases, our new mesh generation method yields larger numbers of enriched terms. This result shows that the new mesh generation approach and pipeline tools we developed are more accurate and can produce statistically more significant results when the number of clusters is large. We also observe that these two methods give similar numbers of cellular component and molecular function terms in all cases. Since the numbers of enriched terms in these two categories are relatively small, the differences in mesh generation methods might not be significant enough to be reflected in these two categories.

**Table 1:** The numbers of enriched gene ontology terms generated by the original (Original) and the proposed (New) mesh generation methods

**Number of clusters**	**Biological process**		**Cellular component**		**Molecular function**	
	**New**	**Original**	**New**	**Original**	**New**	**Original**
30	168	169	36	36	43	43
31	168	169	36	36	43	43
32	155	156	35	35	38	38
33	174	175	30	30	40	40
34	174	175	30	30	40	40
35	169	170	30	30	38	38
36	189	176	30	29	38	38
37	189	176	30	29	38	38
38	189	176	30	29	38	38
39	192	177	32	31	38	38
40	192	177	32	31	38	38
41	222	209	19	18	24	22
42	227	209	20	19	29	22
43	232	209	21	19	28	22
44	231	209	27	19	28	22
45	234	209	28	19	33	22
46	234	209	28	19	33	22
47	234	209	28	19	33	22
48	234	209	28	19	33	22
49	234	209	28	19	33	22
50	228	209	28	19	27	23
51	228	210	28	20	27	23
52	228	221	29	20	27	25
53	228	217	29	20	27	25
54	228	196	29	19	27	25
55	228	196	29	19	27	25
56	228	195	29	22	27	25
57	228	203	29	22	27	21
58	228	204	29	22	28	25
59	228	204	29	23	28	25
60	229	208	29	21	28	26

The number of co-clusters is varied from 30 to 60. In each case, the total number of enriched terms from all clusters are reported.

**Figure 4:**
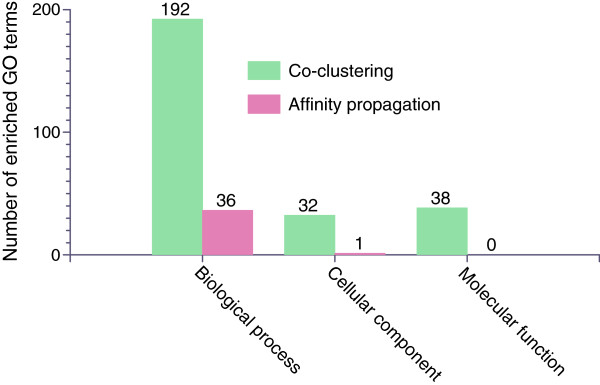
**Comparison of the total numbers of enriched gene ontology terms obtained from our co-clustering method and the affinity propagation method used in **[8]**.** The reported numbers here are the total number of terms in each cluster.

We also compare our co-clustering approach with the affinity propagation method used in [8]. Namely, we compare our EM-based co-clustering method with the affinity propagation clustering by applying these two methods to the data matrix generated by our mesh using 553 images. The affinity propagation method automatically determines the number of clusters and yields 39 clusters on this data set [8]. We also apply our co-clustering method on this data set to generate 39 clusters. We then compute the number of enriched GO terms for each cluster, and the results are depicted in Figure 4. We can see that our co-clustering method is able to generate gene clusters that are functionally more enriched than those by the affinity propagation approach.

The significantly different results might be due to the fundamentally different approaches taken by the two studies. Specifically, Frise *et al*. [8] used clustering method to group the genes into clusters based on all the mesh elements. In another word, clustering method measures the expression patterns of genes across the whole embryo. That is, for two genes to be in the same cluster, they need to have similar expression patterns over the entire embryo. In comparison, we propose to use a co-clustering method, which identifies gene and mesh co-clusters simultaneously. In our approach, two genes can be grouped into the same cluster if they share similar local expression patterns. Note that co-clustering was mainly motivated from gene expression studies [30], and our results show that co-clustering method yields statistically more significant results.

### Co-clustering of mesh elements and genes

We next evaluate the gene co-clusters, and correlate the results with major developmental events occurring during stages 4-6. To accomplish this, we first apply our mesh generation and co-clustering methods to the data set of 2693 images depicting gene expression in stage 4-6 laterally oriented embryos [8]. We set the number of co-clusters to 39 as in [8]. Then, enriched GO terms (biological process) are computed (*p*-value < 0.001). A one-sided significance test is applied, and enriched terms with ≥90% significance were retained. Of the 39 clusters, 21 are enriched in at least one term.

**Figure 5:**
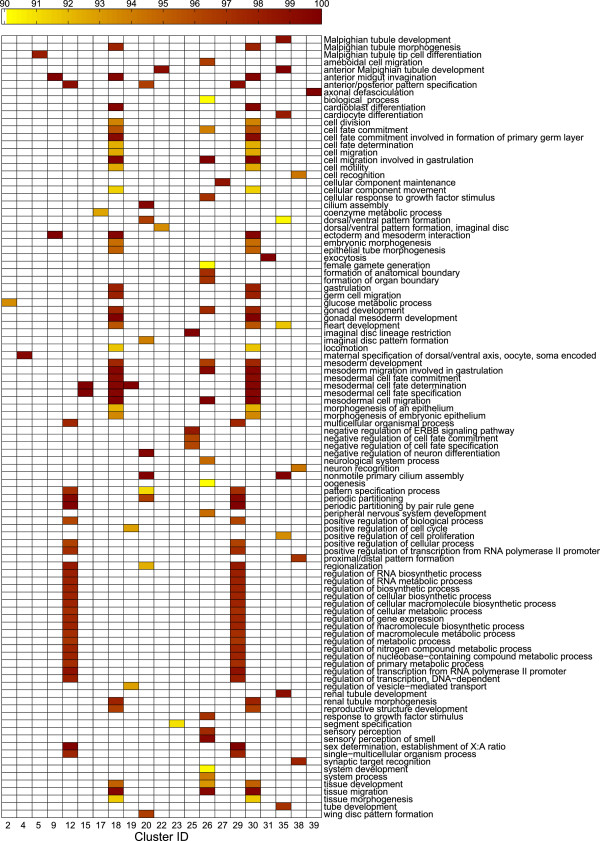
**Significance levels of enriched GO biological process terms on the data set of 2693 images.** Scales indicate significance levels, and only terms with ≥90 significance are shown. A total of 39 co-clusters have been generated, and only co-clusters with enriched terms are shown. The corresponding mesh clusters are shown in Figure 6.

The majority of enriched GO biological process terms are related to gene regulation, embryo development, pattern formation, and cell fate specification (Figure 5). This makes biological sense, as during stages 4-6 cellularization and the start of gastrulation occur. At the beginning of gastrulation, major morphogenetic movements start and the ventral furrow begins to invaginate, ultimately forming the mesoderm as development progresses [4,27]. Of 39 clusters, five (15, 18, 19, 26, and 30) are enriched with terms related to mesodermal cell fate determination and specification, mesoderm development, mesoderm cell migration, and gastrulation.

**Figure 6:**
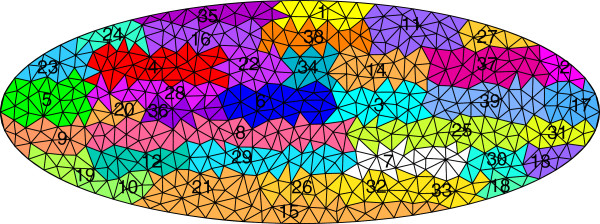
**Mesh clusters corresponding to the gene clusters in Figure **5**.** To establish a correspondence between this figure and Figure 5, each mesh element is labeled with its cluster membership, and the corresponding gene clusters are shown as columns in Figure 5.

Mapping the enriched GO terms back onto the clusters reveals clusters enriched in similar terms are often located in close proximity to each other (Figure 6). For example, looking back at Figure 5, the majority of clusters enriched with terms related to mesoderm development are located in the ventral section of the developing embryo. This location corresponds to the ventral furrow, and many genes verified to be involved in mesoderm specification are expressed in these embryonic regions during stages 4-6 [31,32]. This suggests uncharacterized genes expressed in these domains may be involved in similar developmental processes, and are candidates for experimental testing.

Among the many genes showing expression in clusters located in the forming cephalic furrow, we find a subset of genes known to be involved in the mesodermal developmental network [31,32] among the images in our data set. These include the transcription factors *twist*, *snail*, *Mes2*, *brinker*, and *tinman*. These genes exhibit high co-expression, and are expressed in the ventral region of the embryo during stages 4-6 [31,32]. We obtain similar results when examining other clusters located in close proximity to each other, overall suggesting that the discovered gene and mesh co-clusters correlate well with major developmental events associated with the stage range.

Lastly, we examine the 18 clusters showing no GO biological process enrichment in our stage 4-6 co-clustering. These include clusters 3, 6, 14, 34, and 37. Looking back at the images, we find a lack of localized gene expression at these embryonic domains during stages 4-6. These clusters initially form a single cluster in the interior region of embryo when the number of clusters is small (Figure 2). These regions are involved in later developmental processes and are not involved in the major developmental events occurring during stages 4-6 of *Drosophila* embryogenesis.

## Conclusion

In this study, we aim at identifying co-expressed embryonic domains and the associated genes simultaneously. We develop a mesh generation pipeline that maps the expression patterns of many genes into the same coordinate space. We then employ a co-clustering formulation to cluster the mesh elements and the genes. This identifies co-expressed genes and spatial embryonic domains simultaneously. Experimental results show that the embryonic domains identified in this purely data-driven manner correspond to many embryonic structures. Results also show that the gene and mesh co-clusters correlate with major developmental events during the stages we study.

In the current mesh generation method, we only consider the shapes of embryos when deforming the generic ellipse to each embryo. A more accurate deformation method would take the intensity and texture information of images into account. We will develop more advanced mesh generation method in the future. In this work, we focus on a particular time period of development. We will extend our analysis to multiple stages and employ time-varying analysis in the future [23].

## Competing interests

The authors declare that they have no competing interests.

## Authors’ contributions

SJ conceived the project, WZ, AC, NC, CO, SK, and SJ designed the methodology, WZ, DF, and RL performed the experiments, CO, CK, SN, SK, and SJ interpreted the results, WZ, AC, NC, CK, SK, and SJ drafted the manuscript. All authors have read and approved the final manuscript.
